# Effects of Population Weighting on PM_10_ Concentration Estimation

**DOI:** 10.1155/2020/1561823

**Published:** 2020-04-13

**Authors:** Ameerah Su'ad Abdul Shakor, Muhammad Alfatih Pahrol, Mohamad Iqbal Mazeli

**Affiliations:** Environmental Health Research Centre, Institute for Medical Research, National Institutes of Health, Ministry of Health Malaysia, 40170 Shah Alam, Selangor, Malaysia

## Abstract

Particulate matter with an aerodynamic diameter of 10 *μ*m or less (PM_10_) pollution poses a considerable threat to human health, and the first step in quantifying health impacts of human exposure to PM_10_ pollution is exposure assessment. Population-weighted exposure level (PWEL) estimation is one of the methods that provide a more refined exposure assessment as it includes the spatiotemporal distribution of the population into the pollution concentration estimation. This study assessed the population weighting effects on the estimated PM_10_ concentrations in Malaysia for years 2000, 2008, and 2013. Estimated PM_10_ annual mean concentrations with a spatial resolution of 5 kilometres retrieved from satellite data and population count obtained from the Gridded Population of the World version 4 (GPWv4) from the Centre for International Earth Science Information Network (CIESIN) were overlaid to generate the PWEL of PM_10_ for each state. The calculated PWEL of PM_10_ concentrations were then classified based on the World Health Organization (WHO) and the national Air Quality Guidelines (AQG) and interim targets (IT) for comparison. Results revealed that the annual mean PM_10_ concentrations in Malaysia ranged from 31 to 73 *µ*g/m^3^ but became generally lower, ranging from 20 to 72 *µ*g/m^3^ after population weighting, suggesting that the PM_10_ population exposure in Malaysia might have been overestimated. PWEL of PM_10_ distribution showed that the majority of the population lived in areas that complied with the national AQG, but were vulnerable to exposure level 3 according to the WHO AQG and IT, indicating that the population was nevertheless potentially exposed to significant health effects from long-term exposure to PM_10_ pollution.

## 1. Introduction

Air pollution is a major environmental health threat that poses a complex challenge to public health. Despite various efforts taken to reduce its health impacts, it remains a huge problem due to rapid urbanization and growing population along with economic development. In 2012, an estimated 3.7 million premature deaths worldwide was attributable to air pollution, mainly caused by ischaemic heart disease and stroke, and the remaining 20% of which by respiratory disease [1]. According to the World Health Organization (WHO), Southeast Asia has more than 5% increase of particulate matter (PM) levels between 2008 and 2013. More than 50% of cities and populations in Southeast Asia are experiencing increasing trend of annual PM levels and mostly exceeding the WHO Air Quality Guidelines (AQG) [2].

In Malaysia, notable air pollution sources are from land transportations, industrial emissions, and open burning activities. 70–75% of air pollution in the country are from motor vehicle emissions, while stationary sources of air pollution such as power stations and industrial activities contribute to 20–25% [3]. Malaysia has been experiencing serious haze events since 1994 with more frequent trends over the past few years as reported in the chronology of haze episodes in Malaysia [4]. While open burning of solid wastes and forest fires contributed to only 3–5% of the country's air pollution, it is notable that significant amounts of the air pollution were result of uncontrolled open burning activities from a neighbouring country [3].

Ambient air pollution is a complex mixture of multicomponents. PM and gaseous pollutants such as sulphur dioxide, nitric dioxide, and ozone are among the various components that may affect human health in the short and long term [5, 6]. Among the various components, PM is one of the air pollutants with considerable concern. Studies have found that PM pollution may contribute to adverse pregnancy outcomes [7, 8], type 2 diabetes [9], dementia [10–12], and reduction of life expectancy [13]. And among the different size classes of PM, PM_2.5_ (particulate matter with an aerodynamic diameter less than 2.5 *μ*m) is most consistently associated with adverse health effects [8, 9, 13–15]. However, PM_2.5_ measurement data are presently limited in Malaysia; therefore, analyses in this study were focused on PM_10_ (particulate matter with an aerodynamic diameter less than 10 *μ*m) with larger data availability as opposed to PM_2.5_.

Most PM_10_ exposure assessment studies have used concentrations of pollutants from monitoring stations and assumed that measured concentrations are representative of the mean exposure of the populations [16–18]. However, directly using the ambient air concentrations to directly assess population exposure, without taking into account the disproportionate spatial and temporal distribution of the pollutant and the population, might not give the actual human exposure levels [19]. In addition, studies have found that the actual human exposure levels were in fact different from the mean value due to atmospheric pollutant distribution variations in time and space [20, 21].

Therefore, the aim of this study was to detect the effect of population weighting on PM_10_ concentration estimations. With the spatiotemporal population and pollution distribution variabilities added to the PM_10_ concentration estimations, the level of the pollutant concentrations exposed to the population would be different than the mean PM_10_ concentrations. Population weighting could give a better view of human exposure levels leading to better quantitative assessments which will then provide a stronger basis for critical public health evaluation on the extent of damage caused by PM_10_ pollution.

## 2. Materials and Methods

### 2.1. Site Description

**Figure 1:**
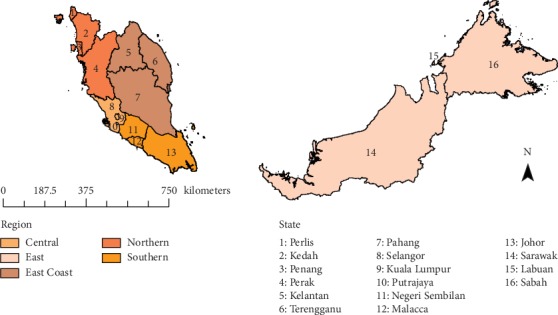
Map of Malaysia and the 5 regions.

Dataset used in this study covered the whole Malaysia for years 2000, 2008, and 2013. Malaysia is a federation consisting of thirteen states and three federal territories [22, 23]. Eleven states and two federal territories (Kuala Lumpur and Putrajaya) are located in the West of Malaysia, which are further classed into 4 regions (Central region, Northern region, Southern region, and East Coast region) in this study, while the remaining two states (Sarawak and Sabah) and one federal territory (Labuan) are classed as East of Malaysia. The three federal territories hereinafter will be referred to as states for simplicity. The 5 regions classified in this study are illustrated in Figure 1.

Putrajaya was afforded Federal Territory status of becoming Malaysia's third Federal Territory in 2001 [23, 24]. The city was developed for the shift of government seat and relocation of federal government headquarters from Kuala Lumpur to Putrajaya because of overpopulation in the former [25, 26]. Prior to the declaration, Putrajaya was a territory entirely enclaved within Sepang, a district in the southern part of Selangor [25]. Hence, Putrajaya's PM_10_ estimate and population count for year 2000 was based on PM_10_ and population data within its geographical coordinates.

### 2.2. PM_10_ Concentration Estimation

The data on aerosol products of Moderate Resolution Imaging Spectroradiometer (MODIS) Terra was obtained from the National Aeronautics and Space Administration's (NASA) Level-1 and Atmosphere Archive and Distribution System (LAADS) Distributed Active Archive Centre (DAAC) website [27]. The aerosol products for MOD04, MOD07, and MOD021 were downloaded for the three studied years at a spatial resolution of 5 km. Data on Aerosol Optical Depths (AOD), surface temperature (ST), atmospheric stability (KI), and relative humidity (RH) were then extracted from the aerosol products and projected to the World Geodetic System (WGS) 84 coordinates using Environment for Visualizing Images (ENVI) software version 5.1. Using the Artificial Neural Network (ANN) model from a local study [28], mean annual PM_10_ concentration estimations were then calculated from the projected outputs in ArcGIS software version 10.1 as follows:

(1) $$\begin{array}{c} {\text{PM}}_{10}=72.599+\left(39.399*\text{H}1\right)+\left(-31.944*\text{H}2\right)+\left(-30.735*\text{H}3\right), \end{array}$$

where H are hidden layers, and

(2) $$\begin{array}{c} \text{H}1\;=\text{ TANH}\left(0.5*\left(\left(-67.612\right)+\left(7.216*\text{AOD}\right)+\left(-0.243\;*\text{ST}\right)+\left(0.214*\text{KI}\right)+\left(0.058*\text{RH}\right)\right)\right),\\ \text{H}2\;=\text{ TANH}\left(0.5*\left(\left(-76.084\right)+\left(3.464*\text{AOD}\right)+\left(-0.319\;*\text{ST}\right)+\left(0.254*\text{KI}\right)+\left(0.057*\text{RH}\right)\right)\right),\\ \text{H}3\;=\text{ TANH}\left(0.5*\left(\left(-32.739\right)+\left(-0.667*\text{AOD}\right)+\left(-0.169\;*\text{ST}\right)+\left(0.114*\text{KI}\right)+\left(0.075*\text{RH}\right)\right)\right). \end{array}$$

**Figure 2:**
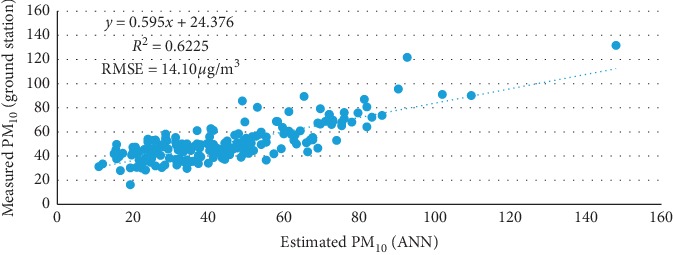
Correlation between PM_10_ concentrations estimations from satellite datasets and measured ground-based PM_10_ concentrations over the years 2000, 2008, and 2013.

The calculated PM_10_ concentration estimations were then spatially interpolated using kriging spatial interpolation to fill in the empty pixels, resampled at a resolution of 0.05° × 0.05°, and validated with ground-based PM_10_ measurements obtained from the Department of Environment Malaysia (DOE). The results obtained in this study were satisfactory (*R*^2^ = 0.6225) between MODIS and the corresponding ground PM_10_ (Figure 2).

### 2.3. Population Distribution Data Collection

Population count was obtained from the Gridded Population of the World version 4 (GPWv4) from the Centre for International Earth Science Information Network (CIESIN). GPWv4 is the latest update of the GPW dataset and it demonstrates the spatial relationship of human population and the environment through gridded data products. Earlier versions of GPW have been extensively used in global population studies [27, 29–31]. Gridded population data with a spatial resolution of 0.05° × 0.05°, matching that of PM_10_ concentration dataset, were downloaded for the years 2000, 2005, and 2010 (Figure 2). Since the GPWv4 provides gridded human population estimations in 5-year intervals starting from year 2000, the gridded population estimates for years 2008 and 2013 in this study were calculated in ArcGIS software using the exponential population projection formula as follows:

(3) $$\begin{array}{c} P_{x}=P_{y}e^{rt}, \end{array}$$

where P_*x*_ is the population estimate in the target year *x*, P_*y*_ is the base population in year *y*, *r* is the average annual Malaysian population growth rate (2.0%) [32], and *t* is the number of years between population counts.

### 2.4. Population-Weighted Exposure Level to PM_10_ Calculation

Using ArcGIS software, the PM_10_ concentration layers were overlaid on the gridded population distribution layers and population-weighted exposure level (PWEL) of PM_10_ for each state were calculated based on the exposure equation as follows:

(4) $$\begin{array}{c} \text{PWEL of }{\text{PM}}_{10}=\frac{\sum \left(P_{i}\times C_{i}\right)}{\sum P_{i}}, \end{array}$$

where P_*i*_ is the population in grid *i* and C_*i*_ is its mean annual PM_10_ concentration.

### 2.5. Population Exposure Levels Categorization

Using the WHO interim target (IT) guideline as the basis [33], five exposure levels were established in this study to categorize the percentage of area and population exposed to different ranges of PWEL of PM_10_ concentration (Table 1). The IT guideline was proposed by the WHO with the intention to reduce mortality risks from exposures to air pollutants by promoting progressive pollutant emission control. Exposure level 5 is when PWEL is greater than 70 *µ*g/m^3^ of PM_10_, suggesting that the population is at more than 15% higher risk for mortality. Lower exposure levels mean more reduction in mortality risks towards the population. Ultimately, exposure level 1 is when the PWEL is less than or equal to 20 *µ*g/m^3^ of PM_10_, meaning that the state has achieved the WHO's AQG for mean annual PM_10_ concentrations. Using ArcGIS software, population exposure distribution maps were then created for visualization of the different PWEL of PM_10_ vulnerability levels in each state during the study period.

Three IT values have been set in the New Malaysia Ambient Air Quality Standard, with an aim of improving air quality in stages and achieving the country's air quality standard by 2020 [34]. With reference to the national standard and IT values, four exposure levels were established in this study to categorize the percentage of area and population exposed to different ranges of PWEL of PM_10_ concentration (Table 2). Between them, level 1 exposure indicates the percentage of land and population that were occupying in areas within the national PM_10_ AQG. Population exposure distribution maps for the study period were then created using ArcGIS software to illustrate the different PWEL of PM_10_ vulnerability levels in each state.

### 2.6. Population Density and Human Exposure Level to PM_10_ Correlation Analysis

Statistical analysis was done to measure the strength and direction of association that existed between population density and PWEL of PM_10_. Inspection of scatter plots between the two variables showed heteroscedasticity. Therefore, Spearman's correlation test was run on the data.

## 3. Results

### 3.1. Malaysian Population Distribution

Table 3 represents the total geographical area, demographic details, and the population densities of each state over the study period. The spatial distribution of population was uneven in each state and population distribution trend was similar for the three years. Population densities were the highest in the Central region, lower in the East Coast region, and the lowest in East of Malaysia. Over the years, Kuala Lumpur remained as the state with the highest population density limited to only 0.07% of the total area for Malaysia, contrary to Sarawak which has the lowest population density despite having the largest area ratio of 37.65%.

### 3.2. PM_10_ Pollution in Malaysia

Table 4 shows the PM_10_ concentrations in Malaysia and its states before and after population weighting. The mean annual pollution concentration in 2013 for the whole country was seen to be increased as compared to 2000. However, lower pollution concentration estimates were observed in 2008 as compared to 2000 and 2008. In general, PM_10_ pollution concentrations were found higher in densely populated states, namely, Kuala Lumpur, Putrajaya, Selangor, and Penang.

### 3.3. Population Exposure to Estimated PM_10_

During the study period, the annual mean PM_10_ concentration and population-weighted exposure levels of PM_10_ in the states ranged from 31 to 73 *µ*g/m³, whereas the latter showed concentrations from 20 to 72 *µ*g/m³ (Table 4). With population weighting, PM_10_ was generally lower than the mean concentration in most states, except in Kuala Lumpur in 2000; Putrajaya, Pahang, and Terengganu in 2008; and Selangor in 2013 where PWEL of PM_10_ were slightly higher than the mean concentration.

### 3.4. Population and Area Ratio in Different Exposure Levels

**Figure 3:**
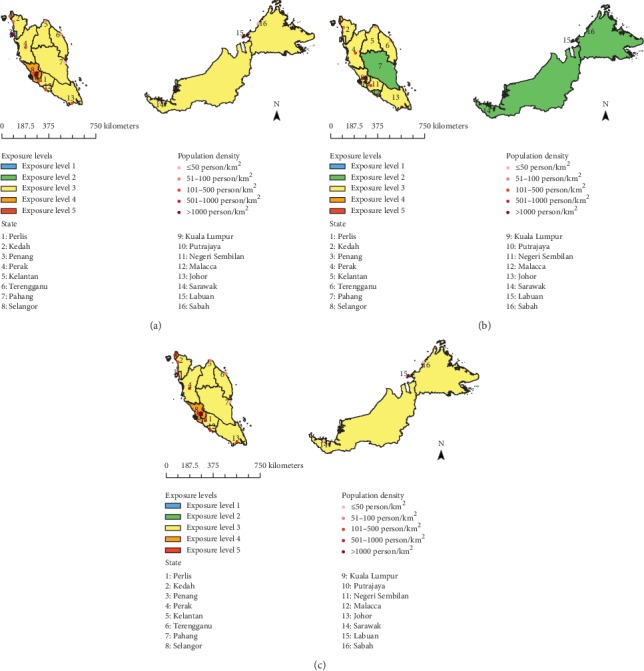
The area distributions of Malaysia in different PWEL of PM_10_ concentration levels according to WHO guideline and interim targets for annual mean PM_10_ concentrations in (a) 2000, (b) 2008, and (c) 2013.

Table 5 and Figure 3 show the percentage of area and population exposed to different ranges of PWEL of PM_10_ concentration with reference to the WHO IT and AQG. During the key period, 65.40–73.38% of the Malaysian population were living in level 3 exposure areas where PWEL ranged from 31 to 50 *µ*g/m³ of PM_10_. In year 2000, only Penang which accounted for 0.31% of the land occupied by 5.05% of the total Malaysian population complied with the WHO guideline for annual mean PM_10_ concentration, whereas in 2008 and 2013, none of the states achieved the WHO recommended mean annual PM_10_ concentration levels. None of the areas were seen to reach level 5 exposure with PM_10_ concentration exceeding 70 *µ*g/m³ in 2000 and 2008. However, population exposure to the pollutant worsened in 2013, where the whole country was above exposure level 2, with Putrajaya accounting for 0.01% of the land and 0.25% of the Malaysian population were vulnerable to level 5 exposure.

**Figure 4:**
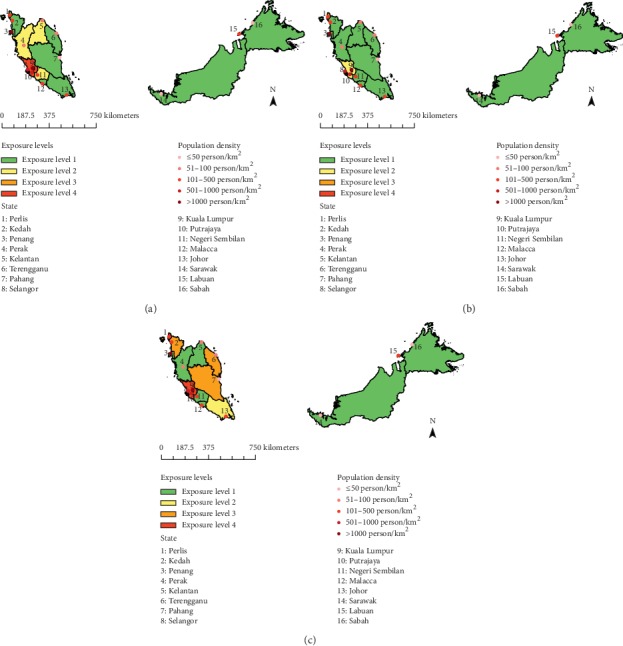
The area distributions of Malaysia in different PWEL of PM_10_ concentration levels according to new Malaysia ambient air quality standard and interim targets for annual mean PM_10_ concentrations in (a) 2000, (b) 2008, and (c) 2013.

Table 6 and Figure 4 show the percentage of area and population exposed to different ranges of PWEL of PM_10_ concentration with reference to the national PM_10_ IT and AQG. A majority of the population were seen living in areas that complied with the national AQG where PM_10_ concentrations were less than or equal to 40 *µ*g/m³. However, the ratio of area and population living in the national recommended mean annual PM_10_ concentration levels decreased in 2013. Less land and less people resided in exposure level 1 and 2 areas, while more land and more people resided in exposure level 3 and 4 areas were observed in 2013 as compared to 2000.

### 3.5. Correlation between Population Density and Human Exposure

Results showed that there was low positive correlation between population density and PWEL of PM_10_, *r* = 0.442, *p* = 0.002 (Table 7), suggesting that the two were marginally correlated. The data reflects that as population density increases in value, so does the PWEL of PM_10_ to some extent. However, the amount of increase was not constant over the whole range of values.

## 4. Discussion

Malaysia is a rapidly developing and newly industrialized country. Industrial activities have polluted the air with smoke from vehicles, factories, and open burning activities; hence, an increasing trend of PM_10_ was assumed as years go by from 2000 to 2013. However, this study revealed an overall reduction of PM_10_ concentrations in 2008. Upon further investigation, it was found that the lower PM_10_ concentrations in between the key period could be partially attributable to local meteorological conditions and confounding factors such as precipitation, humidity, and economy.

Studies have found that influence of climate, particularly rainfall, contributes to lower PM_10_ concentrations through washing effect [35–38]. It should be noted that the mean annual rainfall in Malaysia was higher in 2008 as compared to 2000 and 2013, where the average yearly precipitation recorded for the country was 2393 mm [39], 10535 mm, and 9109 mm [40, 41] for 2000, 2008, and 2013, respectively. Therefore, the higher precipitation level could have reduced the PM_10_ concentrations in 2008. Additionally, the 24-hour mean RH that was extracted and calculated from the downloaded aerosol products revealed that the average RH was 62%, 67%, and 65% for years 2000, 2008, and 2013, respectively [42]. Due to the fact that particulate matter tends to accumulate and deposit on the ground rather than becoming airborne when ambient relative humidity is high, the higher RH index as a consequent of higher rainfall in 2008 could also explain the lower PM_10_ concentration levels in that year. These findings are consistent with several studies that have assessed the linkages between meteorological processes and air pollution and have found that humidity increases as rainfall increases which then subsequently decreases the PM_10_ concentration [37, 38].

Apart from climate, economy may also influence the variation of air pollution concentrations of a country in several ways. It is noteworthy that Malaysia was affected by the Global Financial Crisis in 2008 after a decade of steady economic growth [43]. Censuses have reported that the country had faced declinations in construction, manufacturing, mining, and quarrying businesses in 2008 in line with the deterioration in external demand [44, 45]. Malaysia's economy started to recover the next year and in 2013 the government announced that the country's gross national income per capita then had increased by 50% from 2009 [43]. Similarly, previous studies have also remarked reductions of particulate pollution in relation to economic recessions [46], followed by reversion of the pollution levels after the economy rebounds [3, 47, 48].

One of the variables in the calculation of the population exposure is the distribution of population density, i.e., population in grid *i* (P_*i*_). It is dependent on the dynamics of local populations which could be seen highly influenced by urbanisation. In Malaysia, significant urban agglomeration is observed more towards the central region with an urbanisation level of 100% in Kuala Lumpur and Putrajaya and 91.4% in Selangor. Apart from the three states, another state with high level of urbanisation is Penang in the northern region (90.8%) [49]. When calculating PWEL of PM_10_ for each state, it is expected that the exposure increases as the population density increases. Other exposure studies have reported higher PM_10_ concentrations after population weighting, attributing it to high population density [31, 50–52]. However, results from this study revealed the opposite. Overall, lower PWEL of PM_10_ values were observed in this study indicating that most people were probably exposed to lower PM_10_ concentrations than the actual mean. Despite the contrary, it should be noted that the other studies were done in China and India where the population densities are very much higher as compared to Malaysia. Other than the diverse population and pollution spatial distributions, the massive population density variation between the countries might have also contributed to the differences in postweighting values.

In 2000, Penang unexpectedly recorded the lowest PWEL of PM_10_ concentration for the whole study duration. With a mean annual PM_10_ concentration of 42 *µ*g/m³ and referring to the WHO IT and AQG guideline, Penang should have been categorized in exposure level 3. However, after population weighting, the estimated PM_10_ was lowered to 20 *µ*g/m³, thereby classifying Penang in exposure level 1. Moreover, Penang is one of the country's most urbanised states and accommodates among the nation's highest population densities [49]; thus, higher exposure levels were presumed. Upon further investigation, it was discovered that the state's economic growth slowed down in 2000 due to weakening of manufacturing dynamism [53]. Likewise, previous studies have shown that attenuation of industrial sources of particulate pollution led to pollution reduction [47, 48, 54]. The combination of the lower spatial distribution of PM_10_ on top of the high population density could explain the reduced PWEL of PM_10_ concentration estimations. Therefore, population density and urbanisation level of a region are not the only factors that may reflect PWEL of PM_10_ values, but rather a combination of demographic and local contributing factors.

Though PWEL of PM_10_ concentrations in Malaysia were overall lower as compared to the actual mean, results from this study have shown that the population were nevertheless exposed to worsening levels of PM_10_ concentration. This is regrettably expected that the more areas are being developed, the more people will migrate and reside in these regions susceptible to unhealthy levels of air quality, making them vulnerable to chronic exposure to the coarse pollutant. These findings are consistent with previous population exposure assessment studies which also noted that more people are living in areas prone to higher PM_10_ concentrations [50, 55, 56].

Industrial emission is one of the major sources of air pollution in Malaysia [57], which is an unfavourable outcome of the intense process of urbanisation and industrial development since this country aimed for rapid economic growth to obtain industrial country status by 2020. Due to this phenomenon, Malaysia has to contend with increasing air pollution and has set its own interim targets with regard to the national capability in air quality management. The cut-off value for PM_10_ pollution in the national AQG and IT guideline is higher than most developed countries because the country has yet to complete its industrialization programs.

To further address this issue, the DOE under The Ministry of Energy, Science, Technology, Environment and Climate Change Malaysia (MESTECC) has outlined national and international preventive measures. One of the efforts include enforcing laws that prohibit the public from engaging in open burning [58]. Malaysia has also signed the ASEAN Agreement on Transboundary Haze Pollution in 2002 as an endeavour to tackle the issue [59]. Ratification of the treaty is hoped to start a new dimension in addressing forest and land deforestation issues as well as transboundary haze concerns.

To improve air quality status for the betterment of people's lives, the DOE have also outlined five strategies under the Pelan Tindakan Udara Bersih (Clean Air Action Plan) developed in 2011 [60]. The five strategies are (1) to reduce motor vehicle emissions, (2) reduce air pollution due to land and forest fires, (3) reduce industrial emissions, (4) develop self-efficacy and ability, and (5) strengthen community engagement and awareness. While it may take decades for the outcomes of these strategies to be visible, the efforts must nevertheless be continued. A great example is the Clean Air Act, the law that defines the United States Environmental Protection Agency's (EPA) responsibilities for protecting and improving its air quality, where actions which have been taken to implement the statutes have reduced the key air pollutant emissions since 1990 by 50% [61]. Therefore, it is evident that persistent and consistent action can definitely lead to dramatic improvements of the air quality.

## 5. Limitation

This study has used satellite remote sensing observations for PM_10_ concentration estimations over Malaysia in view of limited number of air pollution monitoring stations coverage for the whole country. Thick cloud covers and satellite coverage gaps are known limitations when using satellite data and due to mid-latitude jet stream instability, extensive cloudiness is a common phenomenon in Southeast Asia [62]. This occurrence reduces the number of cloud-free images available for AOD retrievals [63] which could affect the estimated PM_10_ concentrations and subsequently influence the PWEL of PM_10_ values.

Moreover, this study only provides an overview on the level of Malaysian population exposure to coarse particulate matter. The average time spent outdoors by the population and source-orientated assessments should be accounted for more accurate inhaled concentrations and human exposure levels. Such investigations would enable more precise and comprehensive evaluations for better understanding of the true level of human exposure to air pollution.

## 6. Conclusion

The mean annual population exposure to PM_10_ in Malaysia for 2000, 2008, and 2013 achieved the national AQG but not the WHO standards, indicating that the majority of the Malaysian population lived in the probable risk zones which can induce significant health effects associated with long-term PM_10_ exposures. Geographic, demographic, and local confounding factors contributed to the dynamic spatiotemporal variations of both PM_10_ concentration and population distribution, which then affected the PWEL of PM_10_ estimation concentrations. Therefore, including population weighting into PM_10_ concentration estimations could improve human exposure assessment as it takes these variables into account. The real situation would hence be better reflected and policy makers will be able to properly evaluate and establish appropriate control measures to achieve the lowest possible risk of population exposure to air pollution.

## Figures and Tables

**Table 1:** Five exposure levels established with reference to the WHO AGQ and IT guideline for annual mean PM_10_ concentrations.

Exposure level	PM_10_ ranges (µg/m³)	WHO interim target	Basis for the selected level
1	PWEL ≤20	Air quality standard	These are the lowest levels at which total, cardiopulmonary, and lung cancer mortality have been shown to increase with more than 95% confidence level in response to long-term exposure to PM_2.5_
2	20 <PWEL ≤30	Interim Target-3 (IT-3)	In addition to other health benefits, these levels reduce the mortality risk by approximately 6% [2–11%] relative to the IT-2 level
3	30 <PWEL ≤50	Interim Target-2 (IT-2)	In addition to other health benefits, these levels lower the risk of premature mortality by approximately 6% [2–11%] relative to the IT-1 level
4	50 <PWEL ≤70	Interim Target-1 (IT-1)	These levels are associated with about a 15% higher long-term mortality risk relative to the AQG level
5	PWEL >70	—	—

**Table 2:** Four exposure levels established with reference to the new Malaysia ambient AGQ and IT guideline for annual mean PM_10_ concentrations.

Exposure level	PM_10_ ranges (*µ*g/m³)	Malaysia interim target	Target year to achieve
1	PWEL ≤40	Air quality standard	2020
2	40 <PWEL ≤45	Interim Target-2 (IT-2)	2018
3	45 <PWEL ≤50	Interim Target-1 (IT-1)	2015
4	PWEL >50	—	—

**Table 3:** Demography and area proportion of Malaysia and its states.

Region	State	Area ratio (%)	2000		2008		2013	
			Population ratio (%)	Population density (persons/km^2^)	Population ratio (%)	Population density (persons/km^2^)	Population ratio (%)	Population density (persons/km^2^)
Central region	Selangor	2.40	22.75	658	23.87	823	24.54	949
	Kuala Lumpur	0.07	5.68	5375	5.66	6376	5.61	7095
	Putrajaya	0.01	0.20	911	0.23	1266	0.25	1562
Northern region	Penang	0.31	5.05	1124	5.07	1345	5.06	1505
	Perak	6.36	8.75	95	8.29	108	7.98	116
	Kedah	2.85	6.60	161	6.42	186	6.30	205
	Perlis	0.24	1.06	306	0.77	263	0.73	281
Southern region	Johor	5.80	11.10	133	11.13	159	11.16	179
	Negeri Sembilan	2.01	3.68	127	3.56	146	3.50	161
	Malacca	0.52	2.45	328	2.54	406	2.59	463
East Coast region	Pahang	10.88	5.31	34	5.05	38	4.88	42
	Terengganu	3.93	3.73	66	3.50	74	3.35	79
	Kelantan	4.57	5.38	82	5.03	91	4.80	97
East Malaysia	Sarawak	37.65	8.70	16	8.35	18	8.14	20
	Sabah	22.36	9.41	29	10.38	38	10.96	46
	Labuan	0.03	0.16	395	0.15	453	0.15	493
Malaysia		100.0	100.0	9840	100.0	11791	100.0	13293

**Table 4:** Mean annual PM_10_ concentration and population-weighted exposure levels of PM_10_ in Malaysia and its states (unit *µ*g/m³).

Region	State	2000		2008		2013	
		Mean PM_10_	PWEL of PM_10_	Mean PM_10_	PWEL of PM_10_	Mean PM_10_	PWEL of PM_10_
Central region	Selangor	53	51	41	44	51	55
	Kuala Lumpur	65	67	56	49	70	57
	Putrajaya	67	33	54	58	73	72
Northern region	Penang	42	20	50	36	55	38
	Perak	48	44	36	32	46	40
	Kedah	47	37	46	39	53	50
	Perlis	43	29	48	46	67	54
Southern region	Johor	47	39	36	32	53	41
	Negeri Sembilan	47	44	37	38	54	40
	Malacca	49	38	40	27	51	36
East Coast region	Pahang	42	38	32	30	50	46
	Terengganu	43	37	35	36	49	49
	Kelantan	42	41	33	36	58	40
East Malaysia	Sarawak	41	36	31	30	39	34
	Sabah	42	35	33	29	40	34
	Labuan	42	26	43	43	45	46
Malaysia		47	38	41	38	53	46

**Table 5:** Exposure levels of the Malaysia population and area distributions in different PWEL of PM_10_ concentrations according to the WHO guideline and interim targets for annual mean PM_10_ concentrations.

Exposure level	PM_10_ ranges (*µ*g/m³)	2000		2008		2013	
		Area ratio (%)	Population ratio (%)	Area ratio (%)	Population ratio (%)	Area ratio (%)	Population ratio (%)
1	PWEL ≤20	0.31	5.05	0.00	0.00	0.00	0.00
2	20 <PWEL ≤30	0.24	1.06	71.41	26.30	0.00	0.00
3	30 <PWEL ≤50	96.97	65.40	28.58	73.38	97.27	68.87
4	50 <PWEL ≤70	2.47	28.41	0.01	0.23	2.71	30.88
5	PWEL >70	0.00	0.00	0.00	0.00	0.01	0.25

**Table 6:** Exposure levels of the Malaysia population and area distributions in different PWEL of PM_10_ concentrations according to the new Malaysia ambient air quality standard and interim targets for annual mean PM_10_ concentrations.

Exposure level	PM_10_ ranges (*µ*g/m³)	2000		2008		2013	
		Area ratio (%)	Population ratio (%)	Area ratio (%)	Population ratio (%)	Area ratio (%)	Population ratio (%)
1	PWEL ≤40	84.55	53.54	97.24	69.27	73.79	43.03
2	40 <PWEL ≤45	12.98	17.96	2.43	24.00	5.80	11.16
3	45 <PWEL ≤50	0.00	0.00	0.31	6.42	17.69	14.68
4	PWEL >50	2.47	28.41	0.01	0.23	2.73	31.13

**Table 7:** Spearman's correlation coefficients *r* of population density and PWEL of PM_10_ (*n* = 48).

Variable	Median (IQR)	*r*	*p* value^a^
Population density (persons/km^2^)	170 (537)	0.438	0.002
PWEL of PM_10_ (*µ*g/m³)	39 (11)		

*Note.* ^a^Spearman's rank order correlation. IQR: interquartile range.

## Data Availability

The data used to support the findings of this study are available from the corresponding author upon request.
